# Low-Pass Image Filtering to Achieve Adversarial Robustness

**DOI:** 10.3390/s23229032

**Published:** 2023-11-07

**Authors:** Vadim Ziyadinov, Maxim Tereshonok

**Affiliations:** 1 Science and Research Department, Moscow Technical University of Communications and Informatics, 111024 Moscow, Russia; m.v.tereshonok@mtuci.ru; 2 Skobeltsyn Institute of Nuclear Physics (SINP MSU), Lomonosov Moscow State University, 119991 Moscow, Russia

**Keywords:** adversarial attacks, artificial neural networks, robustness, image filtering, convolutional neural networks, image recognition, image distortion

## Abstract

In this paper, we continue the research cycle on the properties of convolutional neural network-based image recognition systems and ways to improve noise immunity and robustness. Currently, a popular research area related to artificial neural networks is adversarial attacks. The adversarial attacks on the image are not highly perceptible to the human eye, and they also drastically reduce the neural network’s accuracy. Image perception by a machine is highly dependent on the propagation of high frequency distortions throughout the network. At the same time, a human efficiently ignores high-frequency distortions, perceiving the shape of objects as a whole. We propose a technique to reduce the influence of high-frequency noise on the CNNs. We show that low-pass image filtering can improve the image recognition accuracy in the presence of high-frequency distortions in particular, caused by adversarial attacks. This technique is resource efficient and easy to implement. The proposed technique makes it possible to measure up the logic of an artificial neural network to that of a human, for whom high-frequency distortions are not decisive in object recognition.

## 1. Introduction

Convolutional neural networks (CNNs) are used in a wide range of applications in modern computing since they allow for the automation of a wide class of tasks, such as image classification and segmentation [1], object detection and tracking in video streams [2], and image generation [3,4]. In addition, CNNs are the most effective machine learning tool for some audio processing tasks [5,6]. Recently, an increasing part of computational processing power has been involved in multimedia processing. The growth of overall computing power allows for the use of increasingly complex and demanding machine learning algorithms. The CNNs also allow for the extraction of features from multimedia efficiently and to process big data, so they are used to solve difficult-to-formalize or fuzzy tasks.

However, a significant unsolved problem for CNNs is their sensitivity to distortions and noise. Neural networks trained using clean data do not provide sufficient generalizability to recognize distorted or noisy images. So far, the precise noise/distortion robustness characteristics of CNNs are not known yet, and only a few studies in this field are available [7,8,9]. The adversarial distortions severely reduce the image recognition accuracy since they are targeted to the exact neural network model. One of the first mentions of this problem is the study [10], which demonstrated, among other limitations, the weaknesses in the neural network’s generalization ability. The authors have also found out that adversarial distortions are relatively effective for a variety of neural networks with a diverse number of layers, various architectures, or that have been trained using different datasets. Adversarial images are also transferable to other neural networks, even if these networks are trained with different hyperparameters or datasets. Later, a range of techniques for generating adversarial examples were proposed, including the Fast Gradient Sign Method (FGSM) [11], Deepfool [12], One-pixel attack [13,14], and many others. The maxout network [15], initially achieving an error probability of 0.45%, after the application of FGSM, misclassified 89.4% of adversarial examples, with an average confidence rate of 97.6%. Moreover, with a higher image resolution, the recognition error of adversarial examples increases. Currently, the “arms race” of adversarial attacks and countermeasures is relevant [16,17,18]. There are still no effective methods to counteract the high-frequency adversarial attacks. The autoencoder techniques help in detecting high-frequency adversarial attacks, not mitigating them.

Numerous digitally presented natural images also have distortions. These distortions are usually induced during the imaging process. Such distortions emerge in the images without the attacker’s involvement (unusual camera angles and perspectives, camera matrix thermal noise and lens features, atmospheric distortions, image digitization, and compression artefacts). Natural adversarial examples are unpredictable, so the corresponding mitigation methods are often not obvious.

**Figure 1:**
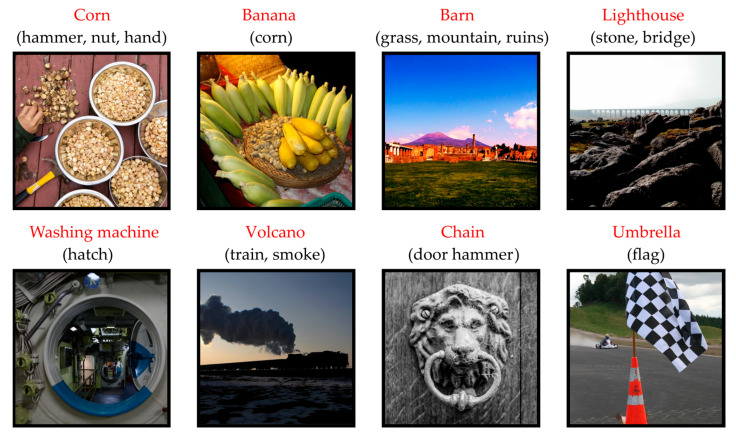
Examples of natural adversarial images from ImageNet-A dataset. The black text shows the actual image class, and red text shows the result of recognition using ResNet-50.

These distortions are referred to as domain shifts [19] and can be exploited by attackers [20]. One of the first works on natural adversarial examples is [21]. Based on the ImageNet dataset, which includes tens of millions of images, the authors created datasets (ImageNet-A and ImageNet-O) containing images that are the worst recognized by the state-of-the-art machine learning models. At the same time, the presented images contain a limited number of false features (Figure 1).

State-of-the-art convolutional network models such as AlexNet, DenseNet-121, ResNet-50, SqueezeNet, and VGG-19 achieve a recognition accuracy no higher than 2.2% on the ImageNet-A dataset (which is approximately 90% lower than the recognition accuracy of the ImageNet dataset by the same networks). The work [21] shows that existing data augmentation methods do not improve performance significantly. Training on other public datasets provides limited improvement. However, [21] does not propose efficient ways to overcome the effect of adversarial distortion. The above-mentioned problems must be addressed in developing modern CNN-based image recognition systems.

Some works that focused on mitigation methods to cope with distortions and noise in images are known [22,23,24,25,26,27]. Some of these works propose various denoising filters, i.e., image preprocessing, generative adversarial networks, and training with noisy data. Most image preprocessing systems are specific to certain types of distortions and adversarial attack designs, so they are being quickly overcome by new adversarial algorithms [28,29]. Important requirements for denoisers, such as boundaries and texture preservation, do not give an advantage in resisting adversarial attacks.

Another known technique to provide adversarial robustness is to use two or more opposing networks. Here, a competing adversarial network generates distorted images to provide misclassification by the classifier. The classifier is trained to resist these attacks [30,31]. Accordingly, adversarial examples can be a good source of augmentation. This augmentation method is effective for increasing the CNN robustness to unobvious and unobservable distortions. However, this approach significantly complicates the development process, the neural network training, and also requires training process monitoring, and is still not always reliable [32]. A crucial way to counteract noise and distortion in test data is to train a neural network using augmented data [33,34]. Various methods, specific to the task, are used for data augmentation. However, a significant amount of research related to CNNs application still does not address this problem.

We can summarize the known adversarial noise countermeasure methods as follows:

1. Defensive distillation [24] implies using two or more networks; it is good for some undefined threats, but weak against fine-tuning the high-frequency attacks;
2. Gradient regularization [35,36]—it is hard to implement; no quantitative evaluation for gradient-based attack robustness is available;
3. Denoisers—they are used mostly for visual image enhancement or upscaling, not proven to be effective against gradient-based attacks; little quantitative evaluation is available [26];
4. There is a work implementing a generator for synthesizing images [37], its authors use incomparable CNN model and datasets;
5. Generative adversarial networks [27] are effective for detecting adversarial noise; the discriminator (the important part of GANs) is also vulnerable to the same adversarial attacks;
6. Low-level transformations [38] are easy and effective techniques. Still, available results are incomparable (different CNN model and datasets).

**Figure 2:**
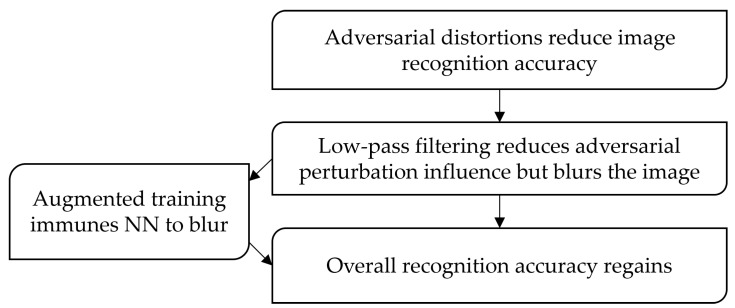
The essence of the proposed technique.

In this paper, we propose a technique to reduce the influence of high-frequency noise on the CNNs. We adopt radio engineering principles—filtering noisy images using a low-pass Gaussian filter [39,40]. Image filtering allows for the suppression of high-frequency noise. In addition, the filtering blurs the image, reducing its sharpness. This leads to a recognition accuracy decrease, as a CNN is initially trained to recognize sharp images. Thus, filtering images with a Gaussian filter allows us to reduce the problem of overcoming high-frequency adversarial attacks to the problem of blurred image recognition, considered in our previous work [41]. We perform a large set of tests for FGSM intensities and Gaussian filter sizes. This allows us to determine the optimal Gaussian filter size for the proposed technique. The essence of the proposed technique is shown in Figure 2. We do not consider complex image preprocessing systems, such as GAN or autoencoders since they are not effective against gradient-based adversarial attacks. The proposed technique is easy to implement and efficient. It can be used in various image recognition systems implemented on a variety of hardware platforms, including those with extremely limited computational resources.

To improve the recognition accuracy, it is essential to train the recognition system to recognize blurred images efficiently. It can be completed by training the recognition system using augmentation with blurred images [42]. In this paper, we prove that this technique for image pre-processing effectively improves noisy image recognition accuracy without a significant reduction in clean image recognition accuracy. We show the existence of an optimum for the Gaussian filter size and propose a technique for finding this optimum. We analyze and compare the behavior of two neural networks: a simple convolutional neural network and the state-of-the-art EfficientNetB3 network [43]. Our simple CNN is tested on datasets with a small number of classes, such as CIFAR-10, Natural Images, and Rock-Paper-Scissors datasets. The EfficientNetB3 is tested on Natural Images and ImageNet-1k.

## 2. Materials and Methods

### 2.1. Datasets

To evaluate the performance of the proposed framework under different conditions and confirm transferability, we carried out experiments on publicly available datasets. We used 4 datasets to train the networks and analyze the results, including CIFAR-10, ImageNet, Rock-Paper-Scissors, and the Natural Images datasets. In this subsection we provide the description of these datasets and briefly describe the justification of our choice.

**Figure 3:**
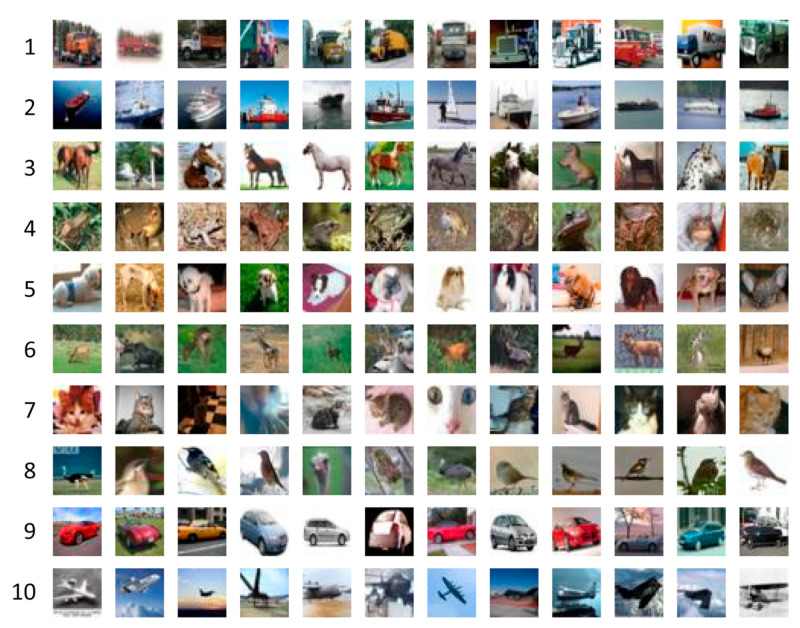
Image examples from the CIFAR-10 dataset (1—truck, 2—ship, 3—horse, 4—frog, 5—dog, 6—deer, 7—cat, 8—bird, 9—car, 10—plane).

CIFAR-10 is one of the most widely used image sets for CNN training and testing. The dataset includes 60,000 images in 10 classes, and the image resolution is 32 × 32 × 3 [44]. This resolution is relatively low, which, on the one hand, allows us to spend much less time and computational resources for training. On the contrary, it significantly reduces the recognition accuracy of distorted or noisy images, even with low noise intensity (Figure 3).

**Figure 4:**
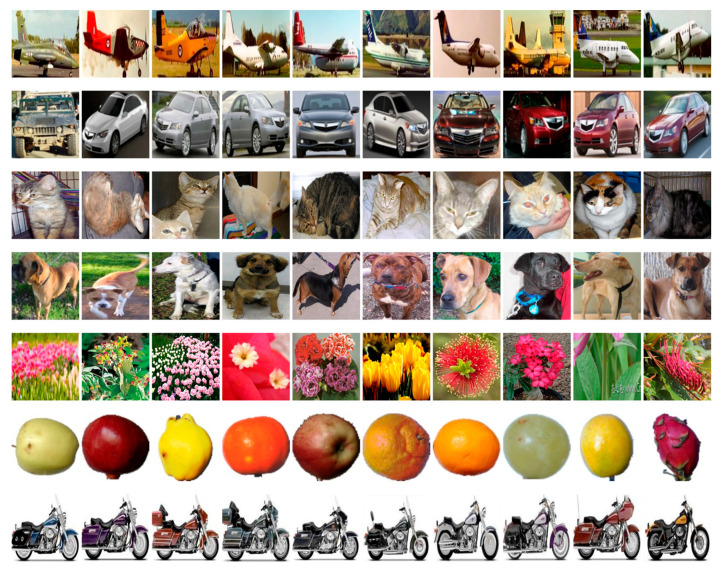
Image examples from the Natural Images dataset.

Natural Images is a comparatively small dataset of natural images [45] consisting of 6899 images of 8 different classes (aircraft, car, cat, dog, flower, fruit, motorcycle, human) (Figure 4). Since training neural networks using large datasets such as ImageNet-1k is challenging, we used the Natural Images set to run a broad class of tests in order to reduce the time and computational cost.

ImageNet-1k [46], a subset of the ImageNet dataset, is a large dataset, containing ~1.4 million images labeled into 1000 classes. The image resolution is not standardized. Images are represented in 3 channels. ImageNet-1k is widely used for testing automated image localization and classification systems, as it has rather complex feature sets and class diversity. We used the ImageNet-1k dataset to extend and validate the results of this research on a complex dataset.

The Rock-Paper-Scissors (RPS) Images dataset [47] contains images of hand gestures from the Rock-Paper-Scissors game. Images are obtained as part of a project [47] to implement a Rock-Paper-Scissors game using computer vision and machine learning. The dataset contains 2188 images corresponding to the gestures “Stone” (726 images), “Paper” (710 images), and “Scissors” (752 images). All images are made on a green background with relatively equal illumination and white balance. All images are RGB with 300 × 200 pixels resolution.

### 2.2. Convolutional Nets

In this study, we used two architectures of convolutional neural networks:

1. Simplified high-speed CNN called SimConvNet; defined below;
2. The commonly used EfficientNetB3 [43].

**Figure 5:**
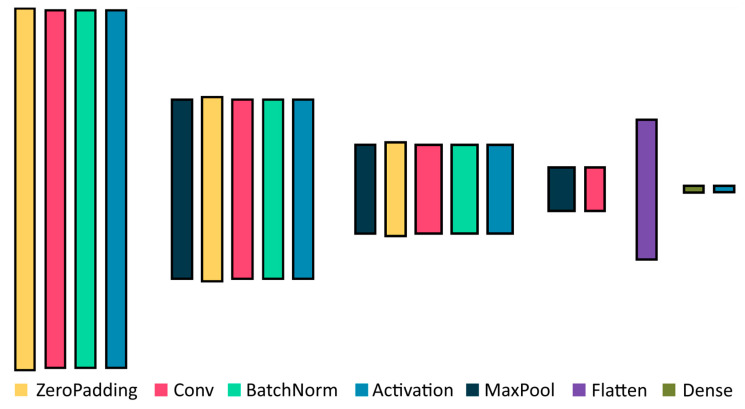
The architecture of simplified high-speed CNN.

We obtained the results of the first experiments using a simplified high-performance network. The network contains 914,960 parameters, which is rather low in comparison to state-of-the-art CNNs. This allows us to conduct brief tests at the expense of overall classification accuracy (Figure 5). The simple CNN is tested on a few small datasets since its generalization ability is extremely limited. We use this simple CNN to confirm transferability of the results to various datasets.

To extend the research and validate results, we used EfficientNet [43]. The research [43] highlighted that insufficient attention is paid to balancing the resolution, width, and depth in the new CNN architectures, and pointed out the importance of such balancing. An efficient method for the combined CNN scaling to any size is proposed in [43]. With orders of magnitude fewer parameters and training time compared to many state-of-the-art network architectures, the EfficientNetB3 architecture achieves higher Top-1 classification accuracy results on various datasets. Since we provide a broad test set in this research, we use EfficientNetB3 to limit the time and computational resources spent on the experiment. It allows us to analyze complex image sets with an acceptable accuracy. The EfficientNetB3 model has enough generalization ability for the complex ImageNet-1k dataset.

### 2.3. Adversarial Attacks

FGSM (Fast Gradient Sign Method) is currently one of the most popular adversarial attack methods [11]. The core idea of the method is to add some non-random vector to the original image. The direction of this vector matches the loss function gradient. The FGSM vector can be represented as:

*η* = *ε*·*sgn*(∇_x_*J*(*θ*,*x*,*y*)),

where *θ* is the neural network model parameters, *x* is the input vector (image), *y* is the true class of vector *x* (if available), *J*(*θ*, *x*, *y*) is the loss function, *ε* is the empirically chosen gain factor, ∇*_x_* is the gradient in image space, *sgn* is a sign function, and *η* is an adversarial vector.

**Figure 6:**
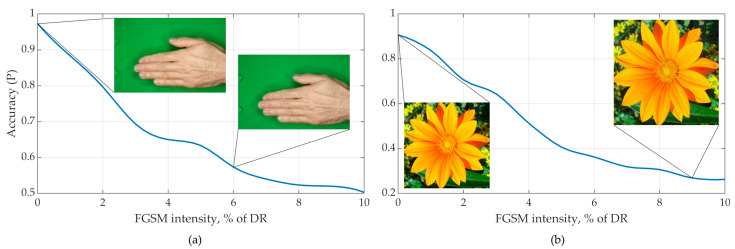
Effect of FGSM on the recognition accuracy of image datasets (**a**) Rock-Paper-Scissors Images and (**b**) Natural Images.

This adversarial vector looks to human perception as a high-frequency, low-intensity noise that does not affect object recognition ability. However, this noise is extremely efficient in reducing object recognition accuracy by neural networks. The intensity of the attack is chosen in order to minimize the visible changes in the image and at the same time to achieve a sufficient attack success rate. It is possible to perform the attack on some state-of-the-art CNN models preserving the non-visibility of changes to a human (Figure 6).

Although FGSM is one of the first adversarial attack algorithms, it is considered one of the most efficient, is simple to implement, and fast. A more complex variant of FGSM is the PGD (projected gradient descent) algorithm. The essence of the PGD algorithm is to iterate the FGSM algorithm to improve the attack efficiency [48]. Many other adversarial attack algorithms are also based on FGSM [49]. We can presume that a proposed high-frequency noise countermeasure technique can be rather effective against high-frequency distortions such as PGD [48], C&W attack [50], Zeroth Order Optimization (ZOO) [51], HopSkipJumpAttack (HSJA) [52], and DeepFool [12]. At the same time, we should note that the proposed technique will not work well against low-frequency adversarial attacks such as physical space attacks [53] and the Square attack [54].

### 2.4. The Theoretical Approach to the Problem Solution

An important feature of image recognition CNNs is the low receptivity to the object’s size. It makes the influence of both low-frequency and high-frequency image components nearly equal. It is the fundamental difference between the functioning of modern CNNs and human perception. The research [55] investigated the impact of various image frequency spectrum components on the CNN. High-frequency image components cause CNNs’ vulnerability to adversarial attacks [55]. Despite that, human vision is immune to high-frequency image components [56]. Some commonly used filters can exacerbate CNNs’ high frequency distortion vulnerability [55]. Additionally, adversarially robust neural networks tend to use smoother gradients in the convolutional kernels (filters) [55].

**Figure 7:**
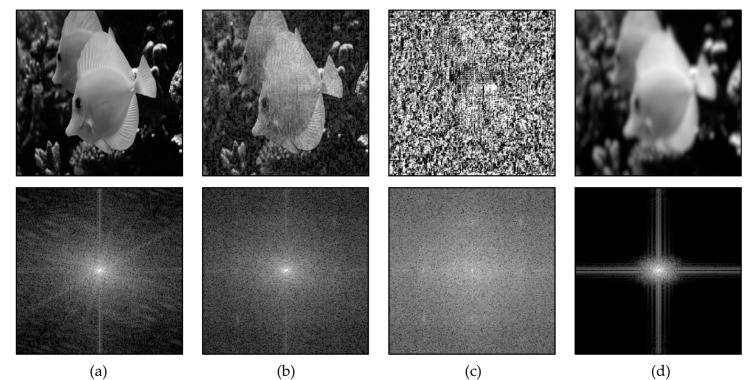
Two-dimensional Fourier transform of an image (**a**) Clean; (**b**) Clean with FGSM (10%); (**c**) FGSM; (**d**) Filtered by Gaussian low-pass filter.

Most adversarial attack algorithms exploit CNNs’ high frequency distortion vulnerability of convolutional neural networks [57]. Some research aimed at detecting the adversarial attacks is based on image spectrum analysis [58,59]. Low-pass filters, such as the Gaussian filter, protect the recognition system from high-frequency distortions, thus being effective in counteracting adversarial attacks. After low-pass filtering, the high-frequency components of the image will be lost, but the overall structure of the image, the position of the objects of interest, and their shapes remain distinguishable (Figure 7).

**Figure 8:**
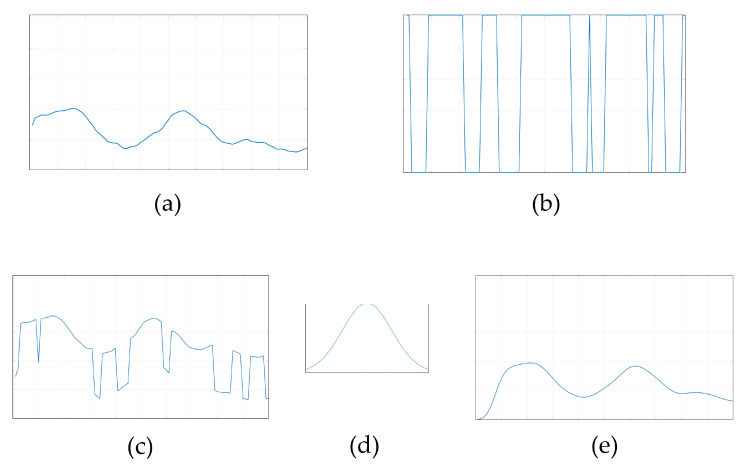
Effect of Gaussian blurring on image content: (**a**) brightness profile of the original image aligned in one line, (**b**) FGSM brightness profile of the same dimension, (**c**) the adversarial image (image + 0.1 FGSM), (**d**) the Gaussian filter impulse response, (**e**) the convolution on the adversarial image and the Gaussian filter impulse response.

Figure 7 shows the Cartesian Fourier power spectrum of the image. FGSM attack erodes the image spectrum. The low-pass filter limits the spectrum, bringing it closer to the original. As another example of reducing the effect of adversarial attacks on an image, we consider it in terms of its images brightness profile. Figure 8 represents the one-dimensional brightness profiles of the image, FGSM 10% of image’s dynamic range, adversarial image, and blurred adversarial image.

**Figure 9:**
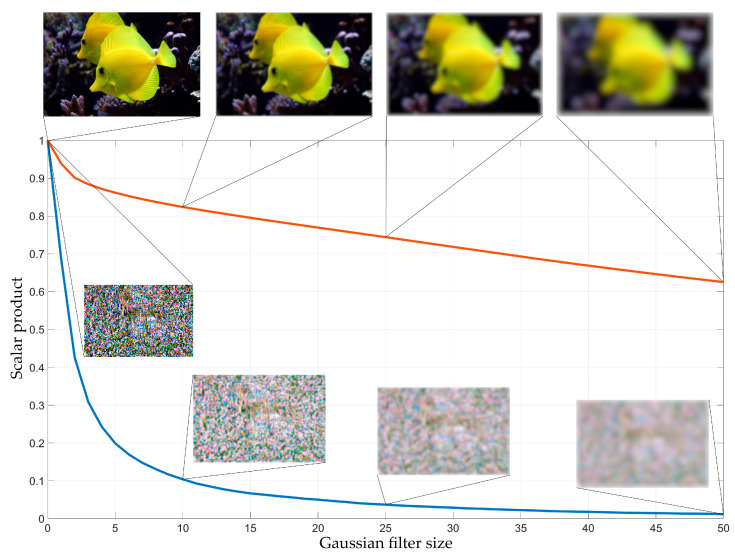
Scalar product of the original image and blurred image (red); the attack matrix and blurred attack matrix (blue) vs Gaussian filter size.

As can be seen in Figure 8, the adversarial attack affects the brightness profile extensively, making it unrecognizable. At the same time, Gaussian filtering made after the adversarial attack restores the brightness profile of the image, bringing it closer to the original one. To confirm the hypothesis about the efficiency of low-pass filtering to overcome the adversarial attack, we analyze the Gaussian blurring effect on the image and attack matrix structure. The red curve in Figure 9 shows the dependence of the scalar product of the blurred and original image on the Gaussian filter size. The blue curve in Figure 9 shows the dependence of the scalar product of the blurred and original attack matrix on the Gaussian filter size. The scalar product of two images (presented as a vectors) can be considered as the similarity or correlation measure. The vectors with similar directions and magnitudes will provide the higher scalar product, and the lower scalar product indicates the orthogonality of vectors.

As one can see in Figure 9, with the Gaussian filter size growth, the scalar product of the original and blurred attack matrix decreases faster than the scalar product of the original and blurred image. With a filter size (standard deviation) exceeding 10 pixels, the blurred and initial attack matrices are nearly uncorrelated. Since the attack matrix is a target function (each pixel is not random), the attack performance will decrease with increasing Gaussian filter size growth more rapidly than the quality of image recognition.

### 2.5. The Proposed Technique

**Figure 10:**
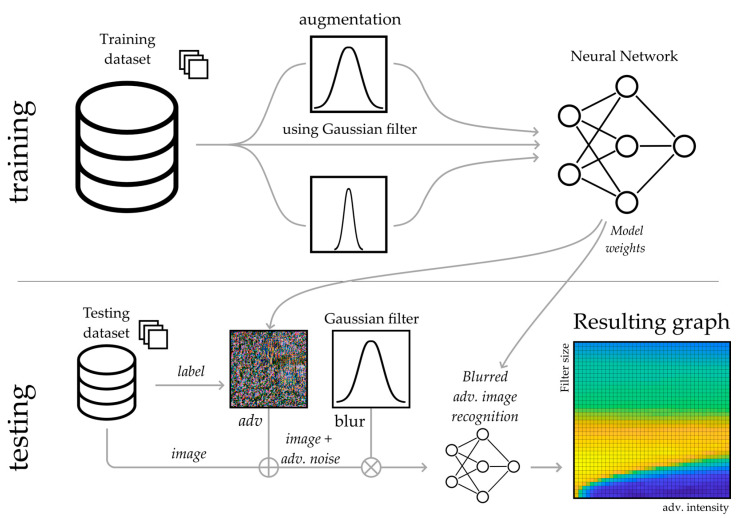
Algorithm scheme.

The block diagram of the proposed image processing algorithm is shown in Figure 10. As for blurring the testing images, it is crucial to train a neural network with blurred data. CNN is pre-trained using the augmented data [42,60]. This approach is efficient since the implementation of a Gaussian filter is computationally cheap. The augmentation procedure uses only this simple filter. The training does not require computationally complex adversarial attack algorithms for data augmentation. We train the neural network in one shot. The original training dataset is split into two parts. One part remained unchanged, the second part was blurred with a filter size chosen randomly in the range between 0 and 0.1 of the image size.

At the testing stage, we added the FGSM vectors to the testing images. After that, the adversarial images were filtered using a Gaussian filter. We used the trained neural network to recognize these blurred adversarial images. The high-frequency image component includes the adversarial attack, other high-frequency noise (e.g., impulse or thermal noise for natural images) and small image patterns. The Gaussian filter significantly reduces the effect of the high-frequency image component. The overall image structure degrades much less significantly. This technique is a trade-off of the overall recognition accuracy for the adversarial image recognition accuracy. The first one decreases just slightly, and the second one rises significantly. We perform a large set of tests involving image recognition with a wide range of FGSM intensities and Gaussian filter sizes. This allows us to obtain 3D plots of the dependence of the image recognition accuracy on FGSM intensity and Gaussian filter size, as well as to determine the optimal Gaussian filter size for recognized images.

## 3. Results

**Figure 11:**
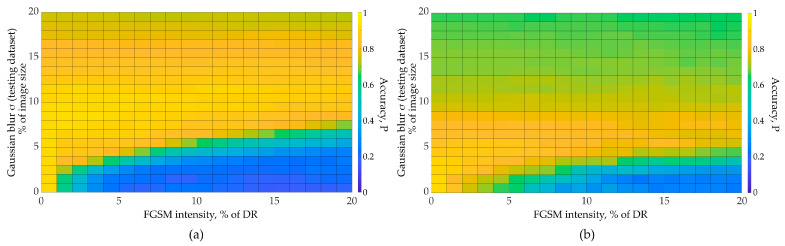
Accuracy for SimConvNet and Natural Dataset: (**a**) CNN trained using augmentation with blurred images; (**b**) no augmentation used.

We obtained the results of the testing dataset recognition for various neural networks using the algorithm presented in Figure 10. The following graphs (Figure 11) show the dependence of image recognition accuracy on FGSM attack intensity and Gaussian filter size. We further evaluate the FGSM attack intensity as a percentage of the image dynamic range (DR). We further evaluate Gaussian filter size as a percentage of the image size.

**Figure 12:**
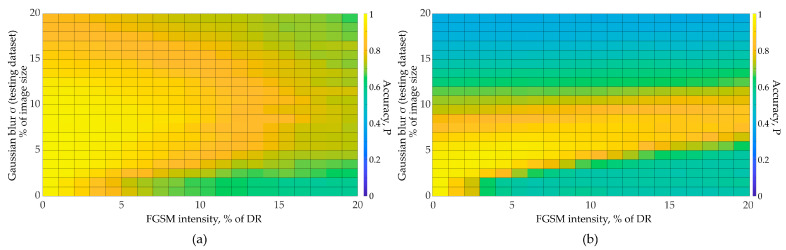
Accuracy for SimConvNet and Rock-Paper-Scissors dataset: (**a**) CNN trained using augmentation with blurred images; (**b**) no augmentation used.

**Figure 13:**
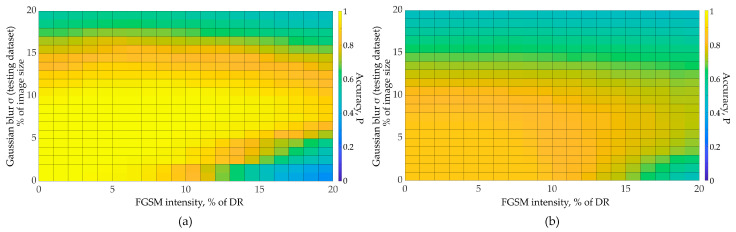
Accuracy for EfficientNetB3: (**a**) Natural dataset; (**b**) ImageNet.

To obtain these graphs, we performed 441 independent experiments on testing dataset recognition with adversarial distortions injection (for each CNN and dataset combination). The total number of independent experiments represented in Figure 11, Figure 12, Figure 13 is 2646. We varied distortion intensities and subsequently processed images with a Gaussian filter. As one can see from the Figure 11, the image recognition accuracy decreases rapidly with increasing adversarial distortion intensity. At the adversarial distortion intensity equal to 4–5% of the image dynamic range (Figure 11a), the recognition accuracy drops to the random level. However, the accuracy increases with Gaussian-filtered adversarial test images. As we further increase the filter size, important image features are lost, and the recognition accuracy drops. Figure 11 shows that as the intensity of adversarial distortion increases, a wider Gaussian filter size is required. Image recognition accuracy does not reach the initial values (as for clean images) but approaches it. With a further increase in the adversarial distortion intensity, the Gaussian filtering becomes less effective. The optimal Gaussian filter size depends on the adversarial distortion intensity, as well as on the features of the data and the neural network, as shown in Figure 11 and Figure 12. For example, CNN with the Rock-Paper-Scissors dataset using augmentation (blurred images) showed high performance at low values of the adversarial distortion intensity (less than 3% of the dynamic range). With a further adversarial distortion intensity increase, the network trained without augmentation obtained a greater gain (Figure 12).

Since, in practice, the intensity of the adversarial attack does not exceed 10–15% of the dynamic range of the original image, the use of image augmentation gives an advantage in recognition accuracy. As one can see from Figure 12, training with an augmented dataset allows us to apply a wider range of Gaussian filter sizes to enhance the recognition accuracy. The obtained results are transferable to complex CNN architectures. In this paper, we conducted experiments using the proposed algorithm (Figure 10) for the EfficientNetB3 using the Natural and ImageNet datasets (Figure 13). We used the augmented ImageNet dataset (augmentation using Gaussian filter). We trained the model without Transfer Learning.

The following table (Table 1) shows the classification accuracy at various adversarial distortion intensities and possible accuracy gain by applying the filter. The optimal filter size was chosen due to the maximization of the recognition accuracy for various values of the adversarial attack intensity.

$$\;\sigma_{opt}=\arg\left(\max\left(\overset{I_{FGSM}^{max}}{\underset{I_{FGSM}=0}{\sum }}P_{LPF}\left(\sigma ,I_{FGSM}\right)\right)\right)$$

where $\;\sigma_{opt}$—optimal filter size, $P_{LPF}$—accuracy achieved using low-pass filtering, $I_{FGSM}$—adversarial attack intensity, $I_{FGSM}^{max}$—maximal adversarial attack intensity. Accuracy gain *G* is calculated using the following formula:

$$G=\frac{\left(1-P_{no\;LPF}\right)}{\left(1-P_{LPF}\right)}$$

where *G*—accuracy gain, $P_{no\;LPF}$—accuracy achieved without use of low-pass filtering, $P_{LPF}$—accuracy achieved using low-pass filtering with optimal filter size. The gain *G* shows the relative drop in the recognition error rate in the case of using low-pass filtering compared to the bare CNN usage.

## 4. Discussion

In this paper, we propose a simple-to-implement method to counteract high-frequency distortions, including high-frequency adversarial attacks. There is still no comprehensive study for the effectiveness of low-pass filtering to counteract high-frequency attacks. The proposed technique can increase the adversarial robustness of deep convolutional neural networks. The method is based on low-pass image filtering and usage of a network trained to recognize blurred images. We show that a Gaussian filter disrupts the adversarial attack structure faster than it blurs the original image features. Thus, the adversarial attack efficiency exchange on the image blurring is found to be efficient. Training the neural network to recognize blurred images is an important part of the proposed technique. This training reduces the impact of image blurring on image recognition accuracy.

The accuracy gain *G* achieved using the proposed technique is in any case not less than 1.4. The average accuracy gain is *G* = 8.8 (excluding EfficientNetB3 evaluated on Natural Dataset and FGSM intensity $I_{FGSM}$ = 5, where the gain is infinite due to the absence of recognition errors with the use of low-pass filtering).

The proposed approach is computationally efficient as it requires only a simple training dataset augmentation performed once before training, and simple image filtering before recognition. The filtering time depends on the resolution of the image. With a simple CNN such as SimConvNet, the time spent on filtering takes less than 0.4% of the overall image recognition time. With complex networks such as EfficientNetB3, the relative time consumption for image filtering is 0.25%.

Several parameters, such as image resolution and neural network type, should be considered when choosing the Gaussian filter size. An excessively high filter size may distort the object features important for classification, thus reducing the overall quality of the neural network algorithm. We show how to choose the optimal filter size.

The proposed method, due to its high efficiency and low complexity, can be used in various image recognition and vision systems implemented on a variety of hardware platforms, including those with extremely limited computational resources. At the same time, we should note that the proposed technique may be ineffective against low-frequency adversarial attacks. In future research, it is expedient to extend the study of the convolutional neural network behavior from the perspective of image preprocessing. This research will include broader sets of state-of-the-art convolutional neural networks, including localization networks. In addition, tests will be provided for the variety of adversarial attacks (BIM, PGD, CW, low-frequency attacks, etc.). A good direction for future research could be the investigation of the effectiveness of the proposed method against the domain shifts. The broader sets of filters, including median filters, rejecting filters, etc., will also be considered.

## Figures and Tables

**Table 1:** Classification accuracy at various adversarial distortion intensities and possible accuracy gain by applying the filter.

FGSM Intensity	FGSM Intensity	$\mathrm{Accuracy}\;\mathrm{with}\;\mathrm{FGSM}\;\mathrm{and}\;\mathrm{no}\;\mathrm{LPF}\;P_{no\;LPF}$	$\mathrm{Accuracy}\;\mathrm{with}\;\mathrm{FGSM}\;\mathrm{and}\;\mathrm{LPF}\;P_{LPF}$	Optimal Low-Pass Filter Size	Accuracy Gain *G*
SimConvNet (Natural Dataset)	5	0.206	**0.913**	10	9.1
	10	0.206	**0.9**		7.9
	20	0.1875	**0.894**		6.7
SimConvNet (RPS)	5	0.738	**0.947**	8	4.9
	10	0.66	**0.879**		2.8
	20	0.576	**0.738**		1.6
EfficientNetB3 (ImageNet)	15	0.699	**0.781**	7	1.4
	20	0.481	**0.72**		1.9
EfficientNetB3 (Natural Dataset)	5	0.977	**1**	7	∞
	10	0.814	**0.996**		46.5
	20	0.25	**0.881**		6.3

## Data Availability

Publicly available datasets were used in this study.

## References

[1] Liu F., Lin G., Shen C. (2015). CRF Learning with CNN Features for Image Segmentation. Pattern Recognit..

[2] Yang L., Liu R., Zhang D., Zhang L. Deep Location-Specific Tracking. Proceedings of the 25th ACM International Conference on Multimedia.

[3] Ren Y., Yu X., Chen J., Li T.H., Li G. Deep Image Spatial Transformation for Person Image Generation. Proceedings of the 2020 IEEE/CVF Conference on Computer Vision and Pattern Recognition (CVPR).

[4] Borji A. (2022). Generated Faces in the Wild: Quantitative Comparison of Stable Diffusion, Midjourney and DALL-E 2. arXiv.

[5] Jasim H.A., Ahmed S.R., Ibrahim A.A., Duru A.D. Classify Bird Species Audio by Augment Convolutional Neural Network. Proceedings of the 2022 International Congress on Human-Computer Interaction, Optimization and Robotic Applications (HORA).

[6] Mustaqeem, Kwon, S (2019). A CNN-Assisted Enhanced Audio Signal Processing for Speech Emotion Recognition. Sensors.

[7] Huang H., Wang Y., Erfani S.M., Gu Q., Bailey J., Ma X. Exploring Architectural Ingredients of Adversarially Robust Deep Neural Networks. Proceedings of the Thirty-Fifth Annual Conference on Neural Information Processing Systems (NeurIPS 2021).

[8] Wu B., Chen J., Cai D., He X., Gu Q. Do Wider Neural Networks Really Help Adversarial Robustness?. Proceedings of the Thirty-Fifth Annual Conference on Neural Information Processing Systems (NeurIPS 2021).

[9] Akrout M. (2019). On the Adversarial Robustness of Neural Networks without Weight Transport. arXiv.

[10] Szegedy C., Zaremba W., Sutskever I., Bruna J., Erhan D., Goodfellow I., Fergus R. (2014). Intriguing Properties of Neural Networks. arXiv.

[11] Goodfellow I.J., Shlens J., Szegedy C. (2014). Explaining and Harnessing Adversarial Examples. arXiv.

[12] Moosavi-Dezfooli S.-M., Fawzi A., Frossard P. DeepFool: A Simple and Accurate Method to Fool Deep Neural Networks. Proceedings of the 2016 IEEE Conference on Computer Vision and Pattern Recognition (CVPR).

[13] Su J., Vargas D.V., Sakurai K. (2019). One Pixel Attack for Fooling Deep Neural Networks. IEEE Trans. Evol. Computat..

[14] Papernot N., McDaniel P., Jha S., Fredrikson M., Celik Z.B., Swami A. The Limitations of Deep Learning in Adversarial Settings. Proceedings of the 2016 IEEE European Symposium on Security and Privacy (EuroS&P).

[15] Goodfellow I., Warde-Farley D., Mirza M., Courville A., Bengio Y. Maxout Networks. Proceedings of the 30th International Conference on Machine Learning.

[16] Hu Y., Kuang W., Qin Z., Li K., Zhang J., Gao Y., Li W., Li K. (2023). Artificial Intelligence Security: Threats and Countermeasures. ACM Comput. Surv..

[17] Chakraborty A., Alam M., Dey V., Chattopadhyay A., Mukhopadhyay D. (2021). A Survey on Adversarial Attacks and Defences. CAAI Trans Intel Tech.

[18] Xu H., Ma Y., Liu H.-C., Deb D., Liu H., Tang J.-L., Jain A.K. (2020). Adversarial Attacks and Defenses in Images, Graphs and Text: A Review. Int. J. Autom. Comput..

[19] Ben-David S., Blitzer J., Crammer K., Pereira F. Analysis of Representations for Domain Adaptation. Proceedings of the Twentieth Annual Conference on Neural Information Processing Systems (NIPS 2006).

[20] Athalye A., Logan E., Andrew I., Kevin K. (2018). Synthesizing Robust Adversarial Examples. PLMR.

[21] Hendrycks D., Zhao K., Basart S., Steinhardt J., Song D. Natural Adversarial Examples. Proceedings of the IEEE/CVF Conference on Computer Vision and Pattern Recognition (CVPR).

[22] Shaham U., Yamada Y., Negahban S. (2018). Understanding Adversarial Training: Increasing Local Stability of Supervised Models through Robust Optimization. Neurocomputing.

[23] Samangouei P., Kabkab M., Chellappa R. Defense-GAN: Protecting Classifiers Against Adversarial Attacks Using Generative Models. Proceedings of the 6th International Conference on Learning Representations, ICLR 2018.

[24] Hinton G., Vinyals O., Dean J. (2015). Distilling the Knowledge in a Neural Network. arXiv.

[25] Xu W., Evans D., Qi Y. Feature Squeezing: Detecting Adversarial Examples in Deep Neural Networks. Proceedings of the 2018 Network and Distributed System Security Symposium.

[26] Liao F., Liang M., Dong Y., Pang T., Hu X., Zhu J. Defense Against Adversarial Attacks Using High-Level Representation Guided Denoiser. Proceedings of the IEEE Conference on Computer Vision and Pattern Recognition (CVPR).

[27] Creswell A., Bharath A.A. (2019). Denoising Adversarial Autoencoders. IEEE Trans. Neural Netw. Learn. Syst..

[28] Rahimi N., Maynor J., Gupta B. Adversarial Machine Learning: Difficulties in Applying Machine Learning to Existing Cybersecurity Systems. Proceedings of the 35th International Conference on Computers and Their Applications, CATA 2020.

[29] Xu H., Li Y., Jin W., Tang J. Adversarial Attacks and Defenses: Frontiers, Advances and Practice. Proceedings of the 26th ACM SIGKDD International Conference on Knowledge Discovery & Data Mining.

[30] Rebuffi S.-A., Gowal S., Calian D.A., Stimberg F., Wiles O., Mann T. (2021). Fixing Data Augmentation to Improve Adversarial Robustness. arXiv.

[31] Wang D., Jin W., Wu Y., Khan A. (2021). Improving Global Adversarial Robustness Generalization with Adversarially Trained GAN. arXiv.

[32] Zhang H., Chen H., Song Z., Boning D., Dhillon I.S., Hsieh C.-J. The Limitations of Adversarial Training and the Blind-Spot Attack. Proceedings of the International Conference on Learning Representations.

[33] Lee H., Kang S., Chung K. (2022). Robust Data Augmentation Generative Adversarial Network for Object Detection. Sensors.

[34] Xiao L., Xu J., Zhao D., Shang E., Zhu Q., Dai B. (2023). Adversarial and Random Transformations for Robust Domain Adaptation and Generalization. Sensors.

[35] Ross A., Doshi-Velez F. (2018). Improving the Adversarial Robustness and Interpretability of Deep Neural Networks by Regularizing Their Input Gradients. Proc. AAAI Conf. Artif. Intell..

[36] Ross A.S., Hughes M.C., Doshi-Velez F. Right for the Right Reasons: Training Differentiable Models by Constraining Their Explanations. Proceedings of the Twenty-Sixth International Joint Conference on Artificial Intelligence.

[37] Li H., Zeng Y., Li G., Lin L., Yu Y. (2020). Online Alternate Generator Against Adversarial Attacks. IEEE Trans. Image Process..

[38] Yin Z., Wang H., Wang J., Tang J., Wang W. (2020). Defense against Adversarial Attacks by Low-level Image Transformations. Int. J. Intell. Syst..

[39] Ito K., Xiong K. (2000). Gaussian Filters for Nonlinear Filtering Problems. IEEE Trans. Automat. Contr..

[40] Blinchikoff H.J., Zverev A.I. (2001). Filtering in the Time and Frequency Domains, revised ed..

[41] Ziyadinov V.V., Tereshonok M.V. Neural Network Image Recognition Robustness with Different Augmentation Methods. Proceedings of the 2022 Systems of Signal Synchronization, Generating and Processing in Telecommunications (SYNCHROINFO).

[42] Ziyadinov V., Tereshonok M. (2022). Noise Immunity and Robustness Study of Image Recognition Using a Convolutional Neural Network. Sensors.

[43] Tan M., Le Q. EfficientNet: Rethinking Model Scaling for Convolutional Neural Networks. Proceedings of the 36th International Conference on Machine Learning.

[44] Krizhevsky A. (2009). Learning Multiple Layers of Features from Tiny Images.

[45] Roy P., Ghosh S., Bhattacharya S., Pal U. (2023). Effects of Degradations on Deep Neural Network Architectures. arXiv.

[46] Russakovsky O., Deng J., Su H., Krause J., Satheesh S., Ma S., Huang Z., Karpathy A., Khosla A., Bernstein M. (2015). ImageNet Large Scale Visual Recognition Challenge. Int. J. Comput. Vis..

[47] Kaggle Rock-Paper-Scissors Images. https://www.kaggle.com/drgfreeman/rockpaperscissors.

[48] Madry A., Makelov A., Schmidt L., Tsipras D., Vladu A. (2017). Towards Deep Learning Models Resistant to Adversarial Attacks. arXiv.

[49] Tramèr F., Papernot N., Goodfellow I., Boneh D., McDaniel P. (2017). The Space of Transferable Adversarial Examples. arXiv.

[50] Carlini N., Wagner D. Towards Evaluating the Robustness of Neural Networks. Proceedings of the 2017 IEEE Symposium on Security and Privacy (SP).

[51] Chen P.-Y., Zhang H., Sharma Y., Yi J., Hsieh C.-J. ZOO: Zeroth Order Optimization Based Black-Box Attacks to Deep Neural Networks without Training Substitute Models. Proceedings of the 10th ACM Workshop on Artificial Intelligence and Security.

[52] Chen J., Jordan M.I., Wainwright M.J. HopSkipJumpAttack: A Query-Efficient Decision-Based Attack. Proceedings of the 2020 IEEE Symposium on Security and Privacy (SP).

[53] Wang J., Yin Z., Hu P., Liu A., Tao R., Qin H., Liu X., Tao D. Defensive Patches for Robust Recognition in the Physical World. Proceedings of the 2022 IEEE/CVF Conference on Computer Vision and Pattern Recognition (CVPR).

[54] Andriushchenko M., Croce F., Flammarion N., Hein M. Square Attack: A Query-Efficient Black-Box Adversarial Attack via Random Search. Proceedings of the 16th European Conference Computer Vision—ECCV 2020.

[55] Wang H., Wu X., Huang Z., Xing E.P. High-Frequency Component Helps Explain the Generalization of Convolutional Neural Networks. Proceedings of the 2020 IEEE/CVF Conference on Computer Vision and Pattern Recognition (CVPR).

[56] Bradley A., Skottun B.C., Ohzawa I., Sclar G., Freeman R.D. (1987). Visual Orientation and Spatial Frequency Discrimination: A Comparison of Single Neurons and Behavior. J. Neurophysiol..

[57] Zhou Y., Hu X., Han J., Wang L., Duan S. (2021). High Frequency Patterns Play a Key Role in the Generation of Adversarial Examples. Neurocomputing.

[58] Zhang Z., Jung C., Liang X. (2019). Adversarial Defense by Suppressing High-Frequency Components. arXiv.

[59] Thang D.D., Matsui T., Vaidya J., Zhang X., Li J. (2019). Automated Detection System for Adversarial Examples with High-Frequency Noises Sieve. Cyberspace Safety and Security.

[60] Ziyadinov V.V., Tereshonok M.V. (2021). Mathematical Models and Recognition Methods For Mobile Subscribers Mutual Placement. T-Comm.

